# Readmission Rates of Patients Discharged against Medical Advice: A Matched Cohort Study

**DOI:** 10.1371/journal.pone.0024459

**Published:** 2011-09-08

**Authors:** Mark Choi, Haerin Kim, Hong Qian, Anita Palepu

**Affiliations:** 1 Department of Medicine, University of British Columbia, Vancouver BC, Canada; 2 Centre for Health Evaluation and Outcome Sciences, St. Paul's Hospital, British Columbia, Canada; Federal University of Rio de Janeiro, Brazil

## Abstract

**Objective:**

We compared the readmission rates and the pattern of readmission among patients discharged against medical advice (AMA) to control patients discharged with approval over a one-year follow-up period.

**Methods:**

A retrospective matched-cohort study of 656 patients(328 were discharged AMA) who were followed for one year after their initial hospitalization at an urban university-affiliated teaching hospital in Vancouver, Canada that serves a population with high prevalence of addiction and psychiatric disorders. Multivariate conditional logistic regression was used to examine the independent association of discharge AMA on 14-day related diagnosis hospital readmission. We fit a multivariate conditional negative binomial regression model to examine the readmission frequency ratio between the AMA and non-AMA group.

**Principal Findings:**

AMA patients were more likely to be homeless (32.3% vs. 11%) and have co-morbid conditions such as psychiatric illnesses, injection drug use, HIV, hepatitis C and previous gastrointestinal bleeding. Patients discharged AMA were more likely to be readmitted: 25.6% vs. 3.4%, p<0.001 by day 14. The AMA group were more likely to be readmitted within 14 days with a related diagnosis than the non-AMA group (Adjusted Odds Ratio 12.0; 95% Confidence Interval [CI]: 3.7–38.9). Patients who left AMA were more likely to be readmitted multiple times at one year compared to the non-AMA group (adjusted frequency ratio 1.6; 95% CI: 1.3–2.0). There was also higher all-cause in-hospital mortality during the 12-month follow-up in the AMA group compared to non-AMA group (6.7% vs. 2.4%, p = 0.01).

**Conclusions:**

Patients discharged AMA were more likely to be homeless and have multiple co-morbid conditions. At one year follow-up, the AMA group had higher readmission rates, were predisposed to multiple readmissions and had a higher in-hospital mortality. Interventions to reduce discharges AMA in high-risk groups need to be developed and tested.

## Introduction

Discharge against medical advice (AMA) makes up 1 to 2% of overall discharges in an acute hospital setting and are known to be higher with certain patient populations [1]–[2]. Earlier studies demonstrated exceedingly high rates of discharges AMA in mental health settings [3]. Several studies in various settings including obstetric, paediatric, general medicine wards and emergency room have also shown that readmission rates of patients discharged AMA are much higher than their counterparts discharged with approval [2]–[10]. Readmission of patients discharged against medical advice leads not only to greater financial burden on the health care system [11]–[13] but also to delays in investigation and treatment of acute illnesses that may lead to higher mortality [1].

Younger age, male sex, poor social support, lack of health care coverage, psychiatric illness, drug or alcohol abuse are frequently associated with discharge AMA [1], [2], [14]–[16]. Two North American studies have examined discharges AMA specifically among general medicine ward patients [7]–[10]. Weingart *et al* showed that patients discharged AMA from the general medicine ward at Beth Israel Hospital in Boston were more likely to be younger, male, and lack health care coverage compared to their counterparts. Not having a primary care physician or admission to hospital with high alcohol level was also associated with discharges AMA [10]. A study based in Toronto, Canada by Hwang *et al* demonstrated significantly higher readmission rates in the AMA group compared with the non-AMA group within the first 15 days. They also showed that up to 40% of patients discharged AMA were readmitted during the first 90 days. History of drug or alcohol abuse was significantly associated with higher rates of discharge AMA [7].

We undertook our study at St. Paul's Hospital, a tertiary care, university-affiliated teaching hospital in Vancouver, British Columbia that serves one of the poorest neighbourhoods in Canada with high rates of addiction, homelessness and HIV [17]. A previous study at this hospital found that among HIV-infected patients, a history of injection drug use was a strong predictor of discharge AMA and more frequent admissions [14].

In this study, we compared the readmission rates and the pattern of readmission among patients discharged AMA to control patients discharged with approval from the general medical and HIV services over a one-year follow-up period. We hypothesized that the rate of hospital readmission would be higher than reported in previous studies given the high prevalence of addiction disorders in our patient population and their increased risk of discharges AMA. These findings may have implications for other hospitals that serve similar marginalized populations in terms of understanding the magnitude of this problem and potential strategies to ameliorate it since often studies of AMA patients are often focused on one chronic physical or mental health condition.

## Methods

### Ethics Statement

The Providence Health Care Research Ethics Board approved this study in December 2009 and because this was a retrospective chart review, they waived the need for patient informed consent.

### Design and Subjects

The study design was a retrospective matched-cohort. We obtained a list of patients discharged against medical advice from either the general medicine or the HIV services between January 1^st^ and December 31^st^, 2008 from the St. Paul's Hospital Health Records. Discharge status (against medical advice [AMA] or with approval) was recorded for all patients by the nursing unit coordinator and included in the medical record. Patients were determined to leave AMA if they did not return to hospital within six hours after obtaining physician permission to go on a pass. The unit coordinator would assign a discharge AMA to the patient if they have not returned by six hours unless a longer period has been authorized by the physician. Patients were also designated as discharge AMA if they simply left the hospital without physician permission and did not return within six hours. Each patient discharged AMA was matched with a patient discharged with approval according to 10-year age group, gender and Case Mix Group (CMG+), which codes for the primary reason for the hospital length of stay [18]. The CMG+ grouping methodology is a case mix tool produced by the Canadian Institute for Health Information (CIHI) to analyze the acute-care inpatient population in Canada. It is designed to aggregate acute care inpatients with similar clinical and resource-utilization characteristics. This methodology, developed using multiple years of acute care inpatient activity and cost records, introduces and enhances several grouping factors to improve the ability to clinically group inpatients and to define length of stay and resource use indicators [18]. If more than one control was eligible for a match, a patient was randomly selected from the list of potential controls. If a patient left AMA on multiple occasions during 2008, we selected the first hospital admission as the index hospitalization. We excluded all discharges where the patient died in hospital.

### Measurement and Analysis

The data were extracted from St. Paul's Hospital electronic medical record database, Sunrise Clinical Manager®. We abstracted patient information including age, gender, housing situation, co-morbidities, medication list, most responsible diagnosis and length of stay for each admission for the 2008 calendar year. The number of readmissions and in-hospital mortality was recorded up to 12 months following the index admission. Housing situation was classified to homeless, single room occupancy hotel, apartment or house. Major categories under co-morbidities included psychiatric illnesses (schizophrenia, psychotic disorder, bipolar disorder, depression and anxiety), neurologic conditions (seizure disorder, stroke, traumatic brain injury), alcoholism, injection drug use status, HIV status, hepatitis C status, congestive heart failure, diabetes, chronic obstructive pulmonary disease, previous gastrointestinal bleeding, and soft tissue infections (cellulitis and abscess). These co-morbidities were selected as these are common conditions seen among patients admitted to the general medical ward at our hospital. They were abstracted from the standardized discharge summary that includes pre-admit co-morbidities and post-admit co-morbidities. The past medical history section of the initial admission note was also reviewed for the above co-morbidities. We created a co-morbidity count indicator variable with three levels: 0–1, 2–3 and ≥4 co-morbidities.

As this was matched case-cohort study using individual matching, we compared baseline characteristics between the AMA and non-AMA group using the McNemar's tests for categorical variables and Wilcoxon signed-rank test for continuous variables. Conditional logistic regression models were fitted to estimate the impact of AMA on the occurrence of hospital readmission related to the index diagnosis. Homelessness, injection drug use, HIV, soft tissue infections, psychiatric illness and the co-morbidity count indicator were included in the model as potential confounding variables. The effect of AMA discharge on the hospital readmission frequency in the one-year follow-up period was evaluated using the conditional negative binomial model adjusting for the aforementioned pre-selected potential confounding variables. Analyses were performed with the SAS software version 9.2. All reported p-values are two-sided.

## Results

Of the 2792 discharges during the calendar year of 2008 from the general medicine and the HIV services, 451 (16.1%) were discharges AMA incurred by 328 patients. Discharge AMA from the general medicine service and the HIV service was 17.8% and 15.4%, respectively. Table 1 presents the demographic characteristics and health care use of the AMA group and its matched non-AMA controls. The AMA group had a higher proportion that were homelessness (32.3% vs. 11%, p<0.001) and had higher median number of Emergency Department visits in the year following their index hospital admission (3 vs. 1, p<0.001). Table 2 compares common medical co-morbidities and socioeconomic characteristics between the AMA and non-AMA groups. Injection drug use (54% vs. 23%, p<0.001), any neurologic condition (22% vs. 7%, p<0.001), alcoholism (28% vs. 13%, p<0.001), history of gastrointestinal bleeding (14% vs. 3%, p<0.001) and any long-term psychiatric illnesses (26% vs. 14%, p<0.001) were significantly more prevalent in the AMA group than in the control group. The median number of co-morbidities were significantly higher in the AMA group compared to the control (4 vs. 2, p<0.001) and more AMA patients had ≥4 co-morbidities than the control group (68.3% vs. 23.5%).

**Table 1:** Patient demographics and health care use in the AMA group and its paired non-AMA group.

Characteristics	AMA (n = 328)	Non-AMA (n = 328)	p-value
Sex			NA
Male	207 (63.1)	212 (64.6)	
Female	121 (36.9)	116 (35.4)	
Mean Age (SD)	42.5 (12.2)	44.4 (12.1)	NA
Age group			
17–29	44 (13.4)	34 (10.4)	
30–39	88 (26.8)	66 (20.1)	
40–49	120 (36.6)	135 (41.2)	
50–59	51 (15.6)	63 (19.2)	
> = 60	25 (7.6)	30 (9.2)	
Housing			<0.0001
Homeless	106 (32.3)	35 (11.0)	
SRO	115 (35.1)	73 (22.3)	
Apartment/house	107 (32.6)	220 (67.1)	
Emergency Department (ED) Visit>10 in past 12 months	28 (8.5%)	17 (5.2%)	0.13
Number of ED visit within 12 months after index admission (median, IQR)*	3 (1, 6)	1 (0, 3)	<0.0001
0	49 (15.1%)	122 (37.5%)	
1–2	96 (29.5%)	95 (29.2%)	
3–5	74 (22.8%)	62 (19.1%)	
6–9	59 (18.2%)	22 (6.8%)	
>10	47 (14.5%)	24 (7.4%)	
LOS of index admission (median, IQR)	4 (2, 9)	7( 4, 13)	<0.0001
0–3 days	138 (42.1)	68 (20.7)	
4–7 days	90 (27.4)	103 (31.4)	
8–14 days	56 (17.1)	83 (25.3)	
≥15 days	44 (13.4)	74 (22.6)	

NA = not applicable as patients were matched on these variables; N (%) unless otherwise indicated; SD = standard deviation; SRO = single room occupancy; LOS = length of stay; IQR = interquartile range.

*Number of missing = 6 (3 pairs) and ED visits are exclusive of those resulting in hospital admission.

**Table 2:** Comparisons of patient co-morbidities between AMA and non-AMA groups.

Patient co-morbidities	AMA(n = 328)	Non-AMA(n = 328)	p-value
Neurologic Conditions	71 (22%)	22 (7%)	<0.001
Alcoholism	93 (28%)	42 (13%)	<0.001
Gastrointestinal bleeding	45 (14%)	11 (3%)	<0.001
Psychiatric illness	86 (26%)	46 (14%)	<0.001
Soft-tissue infections	155 (47%)	75 (23%)	<0.001
HCV	201 (61%)	122 (37%)	<0.001
HIV	111 (34%)	96 (29%)	0.07
Diabetes	19 (6%)	32 (10%)	0.07
COPD	44 (13%)	38 (12%)	0.46
CHF	10 (3%)	11 (3%)	1
Injection Drug Use	178 (54%)	76 (23%)	<0.001
Number of co-morbidities (median, IQR)	4 (3, 5)	2 (1, 3)	<0.001
0–1	31 (9.5%)	134 (40.9%)	
2–3	73 (22.3%)	117 (35.7%)	
≥4	224 (68.3%)	77 (23.5%)	

HCV = hepatitis C virus; COPD = chronic obstructive pulmonary disease; CHF = congestive heart failure.

**Figure 1:**
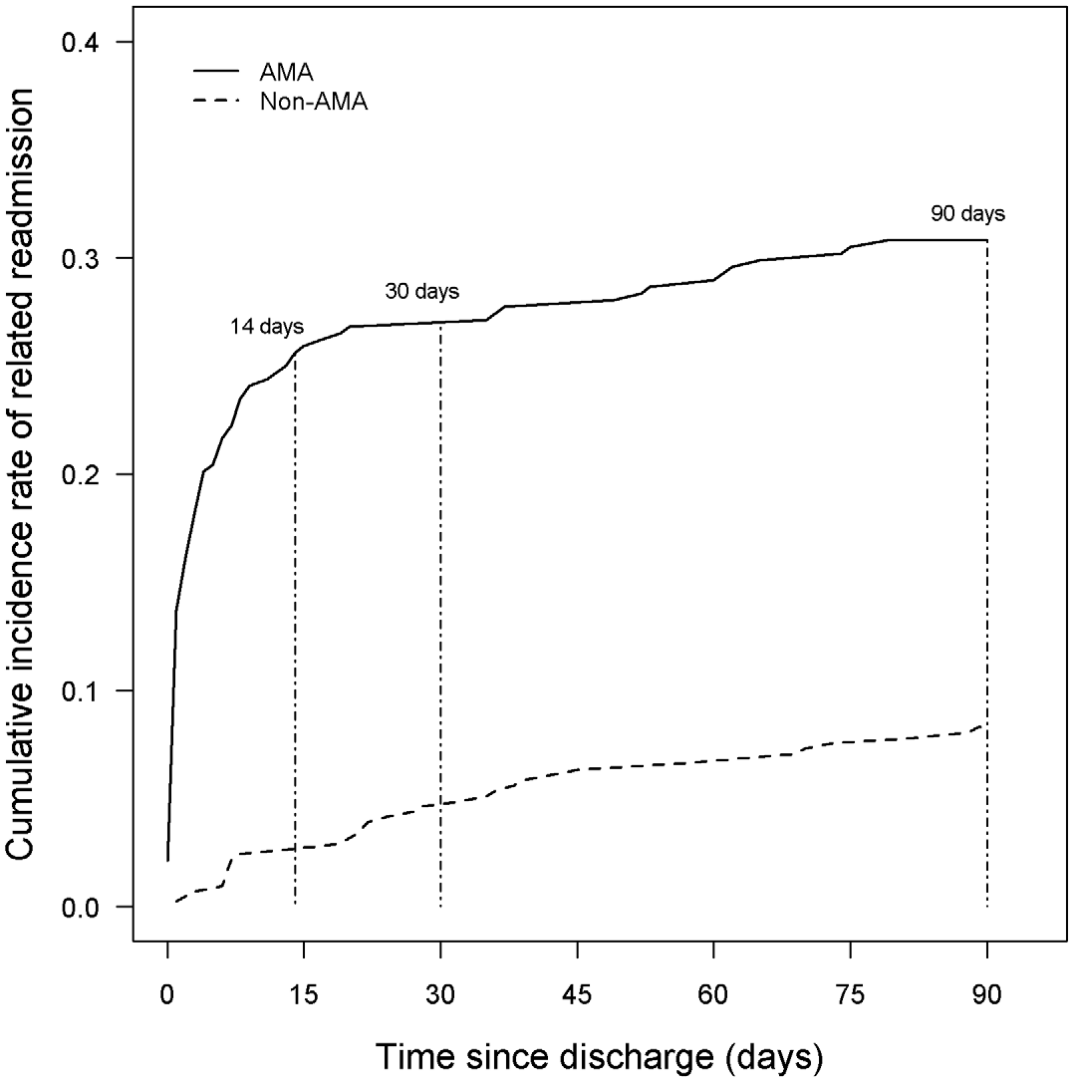
Cumulative hospital readmissions related to index hospitalization diagnosis within 90 days.

Figure 1 represents the cumulative incidence to first readmission within 90 days of the initial discharge by AMA status. Patients in the AMA group had a significantly higher cumulative incidence of readmissions within the first 14 days than the non-AMA group (25.6% vs. 3.4%, p<0.001) and this difference persisted by day 30 (26.8% vs. 5.8%, p<0.001) and by day 90 (30.8% vs. 10.4%, p<0.001). There were seven patients in the AMA group who were readmitted to hospital on the same date of discharge from the index hospitalization; this had a strong effect on the early readmissions as depicted in Figure 1 but did not alter the cumulative incidence of readmission at 14, 30 or 90-days when they were removed from the analysis.

Table 3 presents the unadjusted and adjusted odds ratio for hospital readmission between the AMA and the non-AMA group. Compared to the non-AMA group, the patients discharged AMA were 12 times more likely to be readmitted within 14 days with a related diagnosis (adjusted odds ratio 12.0; 95% Confidence Interval [CI] 3.7–38.9). This significant association relationship persisted at 30-days and at 90-days but was attenuated. With respect to the readmission frequency, patients who left AMA were more likely to be readmitted multiple times at one year compared to the non-AMA group (adjusted frequency ratio 1.6; 95% CI 1.3–2.0). They were also more likely to leave AMA during subsequent readmissions compared to the non-AMA group as depicted in Table 4. We also found higher all-cause in-hospital mortality during the 12-month follow-up in the AMA group compared to the non-AMA group (6.7% [22/328] vs. 2.4% [8/328], unadjusted OR 3.0; 95% CI 1.3–7.1, p = 0.01).

**Table 3:** Conditional logistic regression models hospital readmission related to the index hospitalization diagnosis.

Readmission	Unadjusted OR (AMA vs. non-AMA)		Adjusted OR* (AMA vs. non-AMA)	
	OR and 95% CI	p-value	OR and 95% CI	p-value
Within 14 days	10.1 (4.9–20.9)	<0.001	12.0 (3.7–38.9)	<0.001
Within 30 days	7.9 (4.1–15.3)	<0.001	9.5 (3.3–27.4)	<0.001
Within 90 days	4.4 (2.7–7.1))	<0.001	3.9 (1.9–8.2)	<0.001

*Odds Ratio (OR) adjusted for baseline variables of homelessness, injection drug use, HIV, soft tissue infection, psychiatric illnesses and co-morbidity count indicator variable.

**Table 4:** Distribution of readmissions within one year in the by AMA status.

Number of Readmissions	#1	#2	#3	#4	#5	≥#6
No. of patients (%)						
AMA	187 (57.0)	99 (30.2)	63 (19.2)	36 (11)	24 (7.3)	11 (3.4)
Non-AMA	111 (33.8)	56 (17.1)	26 (9.7)	13 (4.0)	7 (2.1)	2 (0.6)
No. discharged AMA at readmissions (%)						
AMA	82 (43.9)	48 (48.5)	31 (49.2)	12 (33.3)	12 (50.0)	3 (27.3)
Non-AMA	4 (3.6)	2 (3.6)	0	1 (7.7)	1 (14.3)	0

## Discussion

Among hospital discharges from the general medical and HIV services in 2008, we found 16.1% of them were discharges AMA, which is higher than previous reports [1], [10]–[14], [19] and likely reflects the higher prevalence of addiction and homelessness among the patients served by our hospital. We also observed substantial diagnosis-related readmission rates up to 90 days among the AMA group, suggesting that the increased risk of readmission reflects the effect of leaving prematurely with incomplete treatment rather than a high level of co-morbidity among patients leaving AMA. Despite this extensive health care utilization incurred by those who were discharged AMA, we observed a higher in-hospital mortality at one-year.

A 2002 nationwide survey in the United States revealed that 1.4% of all discharges were discharges AMA [2] and a recent study of discharges among Veterans Administration patients over a five-year period found that 1.7% of discharges were AMA [1]. They found that AMA patients were more likely to be black, have low income, have co-morbid alcohol abuse and a higher 30-day readmission rate (17.7% vs. 11.0%, p<0.001) and higher 30-day mortality rate (0.75% vs. 0.61%, p = 0.001) compared to patients discharged with approval. In our study, discharge AMA was much higher than previous studies at 16.1%. This high rate of discharge AMA is likely attributable, but not entirely, to one of the neighbourhoods the hospital serves with its high prevalence of social and economic deprivation [17]. A number of studies have found the following factors to be consistently associated with leaving hospital AMA: lower socioeconomic position, being male, drug and alcohol use, younger age, and in the USA having Medicaid or no health insurance [1], [7], [13], [14], [19]–[22]. Among inpatient psychiatric patients, a comprehensive literature review found that the prevalence of discharge AMA ranged from 3–51% and some of the patient predictors included young age, male gender co-morbid diagnoses of personality or substance use disorders [23]. In our study, the proportion of AMA patients repeatedly leaving AMA during subsequent hospital admissions in the one-year of follow-up remained extremely high (30–50% - Table 4).

Figure 1 shows that most readmissions post-discharge AMA occur within the first two weeks, consistent with previous findings [7], [10]. We found that approximately a third of patients who left AMA were readmitted within 30 days, whereas approximately one-fifth of patients who left AMA in Hwang's study [7] and more than a sixth of patients who left AMA in Weingert's study [10] were readmitted within 30 days. A recent Cochrane systematic review of discharge planning from home to hospital concluded that a structured discharge plan adapted to the patient is associated with small reductions in hospital length of stay and readmission rates for older adults hospitalized with a medical condition [24]. However, the impact of discharge planning on mortality, health outcomes and cost remains uncertain [24].

We also found a high in-hospital all-cause mortality of 6.7% in our study among patients with left AMA during the 12-month follow-up period. Glasgow et al reported a higher 30-day mortality of 0.75% Veterans Administration patients who left AMA vs. non-AMA patients (p = 0.001) over a 5-year period. When they modeled the 60-day mortality, they found a significant association between AMA and mortality (adjusted hazard ratio 1.11; 95% CI, 1.02–1.21) [1]. Two studies of patients admitted to hospital with schizophrenia found a significantly higher mortality due to suicide among those who left AMA [25], [26]. In one of these studies the 60-day mortality rate was 28% among the AMA group compared to the controls (12%), which is alarmingly high [26].

It is unclear how many of the discharges AMA in our sample were potentially preventable. More than half of the subsequent readmissions following an index case of leaving AMA resulted in another discharge AMA as shown in Table 4, suggesting that patients with a history of leaving AMA should be the target of focused interventions to prevent repeat AMA discharges. Suboptimal communication from health care workers and anger over the perceived lack of care that patients receive are common examples of preventable causes of discharge AMA [27]. One recent study explored the reasons for discharge AMA among patient and providers using focus groups [28]. They found a number of themes, many of which related to communication between the providers and patients, in addition to issues of drug addiction and suboptimal pain management [28]. Alfandre summarized a step-wise approach to reducing the rate of discharge AMA [29]. The proposed approach recommends addressing patient's substance abuse, recognizing psychological factors and conducting motivational interviews with the patient [29]. At admission, it may be critical to identify patients at risk of discharge AMA and to begin planning their course of care well before discharge as well as organizing follow-up care during transitions into the community. These findings strongly suggest the need for closely monitoring AMA patients via either telephone consultations or primary physician or nursing visits to review the patient's condition preferably within a week given their co-morbidities [29]. Such approaches may have high potential impact in reducing poor patient outcomes particularly in lower income communities. Our findings highlight the need for more effective health system policies following discharge that span the continuum of care.

This study has several limitations. As a single-site study, our results may not be generalizable to all hospitals but may reflect the situation of other hospitals that serve a substantial portion of indigent patients with significant co-morbidities such as addiction, homelessness and HIV. We did not track patient admissions to other hospitals or ambulatory clinic visits during the one-year follow-up period, or take into account deaths outside the hospital. As a result, the readmission rates reported in this study as well as mortality rate are likely conservative estimates. We did not assess the reliability of the abstracted co-morbidity diagnoses and did not have complete data to use the well-validated Charlson index to adjust for co-morbidity. We were unable to adjust for severity of illness in our analysis due to differences in diagnoses between cases and controls and typically severity of illness measures are disease specific. Furthermore, we did not match the cases and controls for co-morbidity and these between-group differences may contribute to the higher observed mortality in the AMA group. Lastly, we were unable to provide insight regarding the reasons for patients leaving AMA, which are crucial to understand in order to address this significant problem.

In summary, this retrospective matched cohort study found that patients discharged AMA had poor housing situations and multiple co-morbid conditions such as HIV, addiction and psychiatric illness. They had higher readmission rates particularly during the first 14 days post-discharge and were predisposed to multiple readmissions and repeated discharges AMA in the following year. Despite greater health care utilization, the AMA group had poorer health outcomes, most notably higher in-hospital mortality highlighting the importance of developing and testing interventions to reduce AMA discharges in vulnerable populations. A prospective trial is needed to study the effectiveness of interventions such as Alfandre's approach [29] to reducing the rate of discharge AMA among those patients who are at high-risk.
